# Prophylactic and therapeutic DNA vaccines against Chagas disease

**DOI:** 10.1186/s13071-015-0738-0

**Published:** 2015-02-24

**Authors:** Minerva Arce-Fonseca, Martha Rios-Castro, Silvia del Carmen Carrillo-Sánchez, Mariana Martínez-Cruz, Olivia Rodríguez-Morales

**Affiliations:** Department of Molecular Biology, Laboratory of Molecular Immunology and Proteomics. Instituto Nacional de Cardiología “Ignacio Chávez”, Juan Badiano No. 1, Col. Sección XVI, Tlalpan, C.P. 14080 Mexico City, Mexico

**Keywords:** Chagas disease, *Trypanosoma cruzi*, DNA vaccines

## Abstract

Chagas disease is a zoonosis caused by *Trypanosoma cruzi* in which the most affected organ is the heart. Conventional chemotherapy has a very low effectiveness; despite recent efforts, there is currently no better or more effective treatment available. DNA vaccines provide a new alternative for both prevention and treatment of a variety of infectious disorders, including Chagas disease. Recombinant DNA technology has allowed some vaccines to be developed using recombinant proteins or virus-like particles capable of inducing both a humoral and cellular specific immune response. This type of immunization has been successfully used in preclinical studies and there are diverse models for viral, bacterial and/or parasitic diseases, allergies, tumors and other diseases. Therefore, several research groups have been given the task of designing a DNA vaccine against experimental infection with *T. cruzi*. In this review we explain what DNA vaccines are and the most recent studies that have been done to develop them with prophylactic or therapeutic purposes against Chagas disease.

## Introduction

Chagas disease is a zoonosis caused by *Trypanosoma cruzi* in which the most affected organ is the heart. It is estimated that about 10 million people are infected with the parasite in the Americas and 25 million more are at risk of contracting the disease [1]. The effectiveness of conventional chemotherapy is very low; treatment is successful in 55.8% of cases of children with early infection. Most individuals in the chronic phase are resistant to treatment with existing drugs and carry the infection for their lifetime [2-4]. Despite recent efforts, there is currently no better or effective treatment available [5]. DNA vaccines provide an alternative for both prevention and treatment of a variety of infectious diseases, including Chagas disease [6-8].

## Review

### DNA vaccines

In recent years, tools of molecular biology and understanding of the mechanisms of the immune response have helped to expand the development of vaccines. It has been found necessary, in the case of many diseases, to produce vaccines that induce a response by cytotoxic T lymphocytes (CTL). The live attenuated virus vaccines are able to induce both CTL and humoral responses; however, the inactivated virus and the recombinant proteins generally do not induce a potent CTL response. The vaccines from live attenuated organisms provide broad protection, although security for certain diseases may be controversial, such as acquired immunodeficiency syndrome because of the potential risk of reversion to virulence through mutation or recombination with a wild pathogen [9].

Recombinant DNA technology has allowed the creation of some vaccines using recombinant proteins or virus-like particles. These vaccines are considered safe and able to induce an efficient immune response [9,10].

A DNA vaccine can be defined as a plasmid containing a viral, bacterial or parasitic gene that can be expressed in mammalian cells (in the case of infectious diseases) [11,12] or a gene encoding a mammalian protein (in the case of non-infectious diseases such as cancer [13-15], autoimmunity [16-18] and allergies [19-21]). The amount of plasmid that is internalized *in vivo* has been estimated in the picogram range after injection into mouse muscle [22] and in the range of picograms to femtograms after 1 to 7 days of intravenous administration of DNA complexes with cationic lipids [23]. Because the plasmids do not replicate in mammalian cells, the amount available for the gene expression is very low. Therefore, a strong promoter/terminator to drive the expression of the gene must be chosen [9]. Several studies have shown that the amount of antigen produced after inoculation of the DNA is in the range of picograms to nanograms *in vivo*. Plasmid DNA is usually inoculated into the host or animal model by direct injection into the muscle but there are other inoculation routes. The nucleic acid is taken up and expressed by the host cells, causing the subsequent induction of both humoral and cellular specific immune responses [9,10,24]. This type of immunization has been successfully used in preclinical studies and there are diverse models for viral, bacterial and/or parasitic diseases, allergies, tumors and other diseases [24,25]. In recent years, the development of vaccines against parasites has had a significant progress in which a variety of animal models and routes of administration have been tested [26-28]; regarding Chagas disease, several research groups have been given the task of designing a DNA vaccine against experimental infection with *T. cruzi* (Table 1).

**Table 1:** **DNA vaccines containing** ***Trypanosoma cruzi*** **genes for prophylactic use**

**Vector**	**Model**	**Immune Response**	**Survival (%)**	**Recombinant antigen**	**Reference**
pCMVI.UBF3/2	C57BL/6	Th1	30-60	ASP-1, ASP-2 and TSA-1	29
pcDNA3	BALB/c	Th1	100	Clone 9 (ASP-2)	30
pCMV	BALB/c	ND	25-100	TSSA	31
	C57BL/6				
pcDNA3	BALB/c	Th1	ND	catalytic domain of a *trans*-sialidase	32
pcDNA3.1	C57BL/6	Th1	ND	Tc*G1*, Tc*G2* and Tc*G4*	33
pBK-CMV	BALB/c	Th1/Th2	92	TcSP	35
pBK-CMV	BALB/c	Th1	75	TcSSP4	36
pBK-CMV	Beagle dogs	Th1	100	TcSP, TcSSP4	37
pSCREEN	BALB/c	Th1	ND	Cruzipain	40
pcDNA3	C3H/HeN	Th1	100	Cruzipain	41
pCMV	C57BL/6J	Th1	80	LYT1	42
Vaccinia Virus	BALB/c	Th1	25-50	ANYNFTLV epitope	43
pcDNA3.1	BALB/c	ND	70	Tc13	44
pCMV4	BALB/c and C57BL/6 transgenic mice	Th1	75	PFR2 and PFR3	45
pcDNA3.1	Holtzman rats	Th1/Th2	ND	CCL4/MIP-1 beta chemokine	46

ND = Not determined.

### DNA vaccines against Chagas disease

- *Trans-*sialidase family and surface proteins antigens

Garg and Tarleton immunized C57BL/6 mice with ASP-1, ASP-2 and TSA-1 genes of the *trans*-sialidase family and obtained few antibodies and CTL low activity. Protection against lethal challenge was 30% to 60%. When the interleukin-12 (IL-12) and granulocyte macrophage colony-stimulating factor (GM-CSF) genes were co-administered CTL activity, antibody production and resistance to infection with the parasite were increased. Lower parasitism in the tissues analyzed, less swelling and less damage in skeletal muscle were also observed. Survival to the challenge was 60% to 80%; greater protection by the mixture of the three genes was not generated [29].These results show that *trans*-sialidases, remain good candidates for vaccines, but they are a polymorphic gene family and response may vary accordingly. Co-administration of cytokine genes enhances the immune response.

The Boscardin group selected an amastigote surface protein 2 (ASP-2) related-gene of *T. cruzi* which is an antigen recognized by antibodies and T cells from infected mice and humans. Four sets of genes were amplified using specific primers for the *asp-2* gene from amastigotes cDNA of *T.cruzi* Y strain and a clone with the highest degree of identity for the gene of interest was selected, this gene was named *clone 9*. BALB/c mice immunized with a plasmid containing *clone 9* gene produced specific antibodies and CD4^+^ T cells secreting IFN-γ. After trypomastigote challenge a reduction in and increased survival in the animals was observed [30]. The use of parasite surface proteins, secreted proteins or proteins that can be expressed on the infected cell surface are also of interest to identify their possible role as important immunogens for protective induction.

Miyahira and colleagues examined the role of natural killer (NK) T cells against *T. cruzi* infection; they infected CD1d-deficient mice with blood trypomastigotes of Tulahuén strain and they found that the absence of NK cells did not increase susceptibility to infection, comparison of the parasitaemia and survival of these animals with normal BALB/c and C57BL mice being very similar. Subsequently, they analyzed whether administration of α-galactosylceramide (α-GalCer) conferred protection, resulting in a change in parasitaemia only in the late stage of infection, and a slight improvement in survival rate when mice were infected intraperitoneally. The combined use of α-GalCer and benznidazole did not increase the therapeutic efficacy of the drug, suggesting that NK T cells do not play a major role in resistance to infection. In order to determine whether NKcells involved in the immunity induced by DNA immunization, animals were immunized with the pTSSA-plasmid, which encodes a *T. cruzi trans*-sialidase. It was found that parasitaemia was suppressed and survival in infected animals consequently increased in those that were plasmid-immunized, in comparison to animals immunized with the vector alone. Co-administration of α-GalCer and pTSSA-plasmid immunization impaired the protective effect induced by theDNA, as the parasiatemia was increased and did not improve survival compared to mice immunized only with the empty plasmid. When mice were immunized with DNA encoding a CD8^+^ T cells epitope and α-GalCer was co-administered they showed a much lower CD8^+^ T cell production than those animals that were only immunized [31]. This study demonstrates that the induction and activation of NK T cells can have adverse effects on the protective immunity induced by DNA vaccination, possibly because NK cells have Toll-like receptors capable of recognizing parasite-infected cells and probably transfected cells are deleted and thereby the activation of immune response is decreased.

Mussalem et al. used *Propionibacterium acnes* or a soluble polysaccharide extracted from the bacterial wall to modulate the immune response induced by the p154/13 plasmid containing a gene coding for the catalytic domain of a *T. cruzi trans*-sialidase. Treatment with these adjuvants decreased the parasitaemia peak and increased the Th1 specific immune response toward the *trans*-sialidase as was observed by a low IgG1/IgG2a ratio and *in vitro* strong synthesis of IFN-γ by CD4 T cells [32]. These data suggest that adjuvants can improve the DNA vaccines protection against *T. cruzi*.

Bathia and Garg vaccined C57BL/6 mice with three doses of DNA plasmid encoding Tc*G1*, Tc*G2* and Tc*G4* antigens which are expressed in the plasma membrane of trypomastigotes/amastigotes and also with plasmids encoding IL-12 and GM-CSF. IgG2b/IgG1 isotypes were associated with 50%-90% of tissue parasite load control in the acute stage and with an undetectable parasite level during the chronic stage. Serum and cardiac IFN-γ, tumor necrosis factor (TNF) levels and the heart inflammatory infiltrate decreased in vaccinated mice during the development of chronic disease [33]. Together, these results demonstrated the identification of new vaccine candidates that provide protection against *T. cruzi* in experimental mice.

Recently, the Carlos Slim Health Institute proposed an important initiative to accelerate the development of a new bivalent vaccine comprising two *T. cruzi* recombinant antigens, Tc24 and TSA-1, against Chagas disease in Mexico. A consortium including the Centro de Investigación y de Estudios Avanzados of the Instituto Politécnico Nacional (CINVESTAV-IPN), Laboratorios de Biológicos y Reactivos de México, S. A. de C. V. (BIRMEX) in Mexico City, and the Centro de Investigaciones Regionales “Dr. Hideyo Noguchi” in Mérida, state of Yucatán was formed. This initiative will work together with a focus on developing an optimal vaccine in the least possible time [34].

The Rosales-Encina group immunized BALB/c mice with the *TcSP* gene encoding a member of the *trans*-sialidase family, *TcSP4* gene or the recombinant proteins. The serum cytokine analysis showed that immunization with the recombinant protein or with *TcSP* resulted in a T cell mixed Th1-Th2 response. IFN-γ was detected in the *TcSP4* gene vaccinated-mice sera shortly after immunization, suggesting a Th1 response. The immunized mice were infected with the *T. cruzi* H8 strain blood trypomastigotes. Only mice immunized with DNA showed a significant reduction in the peak parasitaemia and lethal challenge survival [35,36]. These studies demonstrate that DNA vaccination induces a protective immune response in contrast to that produced by the homologous recombinant protein during *T. cruzi* experimental infection.

*TcSP* and *TcSSP4* genes were also tested prophylactically in a canine model of Chagas disease; the antibody analysis revealed that the dominant subclass was IgG2. Immunization with both recombinant plasmids induced cell mediated immunity characterized by lymph proliferation and IFN-γ production [37]. In these same animals, immunization decreased the quality and quantity of electrocardiographic changes, thereby preventing progression to more serious cardiac disorders [38]. A partial protective effect for the prevention of macroscopic and microscopic damage in cardiac tissue during the chronic phase was also observed [39]. Two *T. cruzi* genes generating a moderate level of protection in the chronic phase of the disease were proposed.

- Antigenic proteins

Another *T. cruzi* antigen that has been studied by researchers is cruzipain (Cz). It has been shown that this protein is antigenic both in humans and in mice during parasite infection. Schanpp *et al*. immunized mice with the Cz gene and showed a CTL response capable of recognizing and lysing cells infected with *T. cruzi* [40]. Some antigens may be immunogenic and induce protection using recombinant protein or DNA vaccination, such as the Cz gene, which is a good candidate antigen for the development of an anti-*T. cruzi* vaccine. Cz is an enzyme that can be secreted by the parasite and it is considered a virulence factor so if it can be blocked or recognized on the surface of infected cells by CTL then will be a good vaccine candidate, as was observed in this study.

Cazorla *et al.* evaluated mucosal immunization in C3H/HeN mice which received *Salmonella* carrying a plasmid coding for the Cz (SCz) together with bacteria containing a GM-CSF encoding plasmid. As an additional strategy, prime-boost protocols were established in which mice were vaccinated with SCz and were subsequently boosted with recombinant Cz (rCz) co-administered with different adjuvants, such as CpG-ODN and MALP-2 motifs. Protocols of four SCz oral doses induced a mucosal immune response, mainly characterized by the secretion of immunoglobulin A (IgA) and cell proliferation of gut-associated lymphoid tissues, with a weak systemic immune response. In contrast, the protocol including a boost with rCZ and CpG-ODN produced a strong systemic immune response reflected in the titres of specific IgG against Cz, splenocyte proliferation, interferon-gamma (IFN-γ) secretion, and delayed hypersensitivity response. The challenge of vaccinated mice resulted in significantly lower levels of parasitaemia. Parasite control was also evident from the reduction of tissue damage [41]. The results demonstrate that administration of *Salmonella*-mediated Cz-DNA alone or in combination with CpG-ODN or rCz and MALP-2 promotes the immune response to control both the infection with *T. cruzi* and the collateral damage to muscle tissue.

Fralish and Tarleton performed the screening of a cDNA expression library of *T. cruzi* amastigote with monoclonal anti-amastigote antibodies and identified the *FCaBP* gene encoding the flagellar Ca(2^+^) binding protein, Tcβ3 gene encoding a new homologue of the adaptin AP-3 complex β3 subunit and *LYT1* gene which encodes a secreted *T. cruzi* protein involved in cell lysis and infectivity. The three gene peptides induced a response of cytotoxic T cells in chronically infected mice; however, the immunization with only *LYT1* gene protected 80% of mice from *T. cruzi* lethal challenge. Alternatively gene mixtures were tested in immunization assays [42]. This study demonstrates that the ability of *T. cruzi* proteins to induce immune responses in infected hosts, is not necessarily associated with the capacity to induce protection and thus the products of the individual genes and multigene families can serve as effective vaccines.

The Miyahira group used a recombinant adenovirus and the vaccinia virus as vectors expressing a CD8^+^ T cells epitope (ANYNFTLV), which is derived from a *T. cruzi* antigen. The immunization with adenovirus and the vaccinia virus booster was effective in inducing a cellular immune response and consequently protected mice from parasite lethal challenge. The response was increased by co-administration of recombinant vaccinia virus expressing the receptor of the nuclear factor kappa-light-chain-enhancer of activated B cells (NFκB) ligand as adjuvant [43]. The results of this group are spectacular identifying an eight amino acid region of a molecule able to induce a sufficient immune response to control *T. cruzi* lethal infection, but again keep in mind that the peptides to CD4T or CD8T depend on the type of MHC for its proper recognition.

García *et al*. did not find antibodies when DNA plasmid containing the gene of the Tc13 antigen of the *T. cruzi* Tulahuén strain (Tc13 Tul) was administered to BALB/c mice; however, vaccination induced specific memory T cells with no IFN-γ production. From 40% to 80% of the mice immunized showed signs of hepatotoxicity and heart, liver and spleen changes five months post-immunization. After challenge, a significant reduction in parasiatemia during the acute stage was observed without modification in the survival rate. The immunization resulted in decreased severity of cardiac damage in the chronic stage [44]. Although in this study it is reported that the chosen antigen induces protection in the chronic stage of the disease, hepatotoxicity, as well as heart and spleen damage were also demonstrated. It is very important that chosen vaccination antigens are completely safe and they induce a significant level of protection.

Morell *et al.* immunized mice with DNA containing genes for*T. cruzi* paraflagelar rod proteins (PFR2 and PFR3) alone or fused to the 70 kilodalton heat shock protein (HSP70). Both immunizations induced high levels of anti-PFRs IgG2a; however, only the PFR2-HSP70 immunization induced a significant increase in the IL-12 and IFN-γ expression and a decrease in the percentage of cells expressing IL-4 [45]. The chimerical gene immunization may provide a protective response against *T. cruzi* experimental infection.

- Non-specific *T. cruzi* proteins

Roffe *et al*. injected a cardio-toxin and four doses of 100 μg of plasmid encoding for the chemokine (C-C motif) ligand 4/macrophage inflammatory protein 1 beta (CCL4/MIP-1 beta) into rats intramuscularly. After 14 days after the last immunization the rats were challenged with *T. cruzi* CL-Brener clone. The myocarditis was still intense at day 30 but the inflammatory infiltrate showed a focal distribution. Increased anti-CCL4/MIP-1beta levels in the *T. cruzi* infected animals were induced by the immunization. This was associated with an exacerbation of inflammation and fibrosis of the myocardium, although no changes were observed in the myocardial parasitism and parasitaemia rate [46]. This study suggests that CCL4/MIP-1 beta plays a role in preventing excessive inflammation rather than the control of parasite replication.

### DNA vaccines as immunotherapy against Chagas disease

All antigens that have been used in the formulation of therapeutic DNA vaccines belong to the *trans-*sialidase family.

The Sanchez-Burgos group performed intraperitoneal infection in ICR mice with 500 parasites. Five and 12 days later mice were treated with 20 μg of DNA plasmid containing theTSA-1, TS, ASP-2-like, Tc52 and Tc24 antigens. The DNA treatment with the gene encoding Tc52 reduced the parasitaemia and heart parasite load and improved the survival, but myocarditis was not affected significantly. The plasmids encoding Tc24 and TSA-1 significantly reduced parasitaemia, mortality, myocarditis and heart parasite load [47]. These data demonstrate that the therapeutic vaccination efficacy is dependent on the antigen and suggest that DNA vaccines encoding Tc24, TSA-1 and Tc52 represent good candidates for further studies for a therapeutic vaccine against Chagas disease.

Dumonteil *et al.* infected BALB/c mice with lethal doses of parasites and subsequently mice were treated with two immunizations of 100 μg of DNA plasmid containing the Tc24 antigen, starting on day 5 post-infection. The treated animals showed parasitaemia and heart tissue inflammation reduction; the survival was more than 70% with TSA-1 and 100% with Tc24. Parasitological heart tissue analysis indicated that most of the mice contained parasite’s kinetoplast DNA but a few of them showed living parasites, suggesting that the immunotherapy was effective but did not totally eliminate parasites [48]. In this strategy where the infection is already established, the use of therapeutic vaccines may help to reduce the risk of developing the acute stage of infection and therefore the associated pathology, but the parasite cannot be totally eliminated.

In a subsequent study, Zapata *et al.* investigated changes of T cell populations induced by DNA vaccine as immunotherapy. The ICR mice were infected with 500 blood trypomastigotes and treated during the acute or chronic stage with two doses of 100 μg of DNA. Flow cytometry indicated that the therapeutic vaccine induced a rapid increase in the number of CD4^+^ and CD8^+^ T cells in both the acute and chronic stages. Also there was a rapid increase in IFN-γ production by CD8^+^ T cells after treatment for the chronic phase. The effects of these changes in the infection control required long periods of time to be detectable but resulted in a myocarditis and parasite load reduction in both stages of the infection as tested by histopathological analysis and PCR semi-quantitative detection of *T.cruzi* in cardiac tissue [49]. These results suggest that the DNA vaccines inducing CD8^+^ T cell activity and IFN-γ production, as Tc24 gene, might be good candidates for effective therapeutic vaccination against *T. cruzi* infection.

Aparicio-Burgos *et al.* induced an antigen specific IgM and IgG response (IgG2a, IgG1) in dogs after vaccination with TcVac1, which consisted of various plasmids encoding *T. cruzi* antigens, IL-12 and GM-CSF. Increased CD8^+^ T cell proliferation and IFN-γ production as well as suppression of phagocyte activity, were observed. Parasitaemia was controlled and a moderate decrease in the triatomine infectivity was produced by the vaccination; however, heart parasite load and electrocardiographic and histopatologic alterations were not prevented but were less severe than in non-immunized dogs [50]. These results showed a moderate effect on both the acute and the chronic stages of infection but do not avoid the disease.

Rigato’s group used a plasmid and a human adenovirus Type 5 (HuAd5) with a replication defect expressing the *T. cruzi* amastigote surface protein 2 (ASP-2). The aim was to elucidate the immmuno-protective memory T cell phenotype and function. Short and long-term CD8^+^ T cell populations available were compared in detail after immunization. It was found that despite the timing, both populations overlapped greatly with respect to functional and phenotypic characteristics [51]. In a subsequent study it was observed that the up-regulation of CD95 expression and the phenotype of the pro-apoptotic pathogen-specific CD8^+^ T cells expanded during infection were significantly reduced when the adenoviral vaccine was provided at the time of the infection. In parallel, mice vaccinated with adenovirus and subsequently infected had a stronger cell immune response mediated by CD8 T cells and survived an otherwise lethal infection. It was concluded that a suboptimal response of CD8^+^ T cells is associated with up-regulation of CD95 expression and a pro-apoptotic phenotype. Both situations can be blocked by the adenoviral vaccination [52].

Hoft *et al.* tested whether co-administration of a plasmid encoding IL-15 (pIL-15) and a plasmid encoding *trans*-sialidase (pTS) of *Trypanosoma cruzi* could improve the duration of protection, this co-administration did not significantly alter the T cell response or the protection shortly after immunization. However, mice vaccinated with both plasmids and challenged 6 months post immunization were significantly more protected than those who received only the gene of *T. cruzi*. Improved protection correlated with a significant increase of specific IFNγ-producing T cells against *trans*-sialidase [53]. It was shown here that IL-15 may have a significant effect as an adjuvant for induction of increased protection against *T. cruzi.*

Nogueira *et al.* used the yellow fever virus as a vector to immunize mice with the gene encoding the *T. cruzi* ASP-2. It was shown that the recombinant virus YF17D/ENS1/Tc was capable of priming CD8^+^ T cells directed against the *T. cruzi* TEWETG1 epitope producing IFN-γ after challenge [54]*.* This study shows that the use of viral formulations can be a good strategy to induce protective immune responses against pathogens.

## Conclusions

DNA vaccines have provent o be effective in a variety of preclinical models. This technology has been adopted by many researchers as a strategy to develop a suitable vaccine against Chagas disease. It has explored various immunogens (genes encoding a variety of *T. cruzi* proteins), and routes of administration as well as the use of immunomodulators.

Although no vaccine has been able to prevent the infection, in some cases immunizations have induced partial protection; these studies provide data that proves the feasibility of preventive and therapeutic DNA vaccines to control the *T. cruzi* infection. However, a better understanding of the protective immune responses that can effectively stop parasite development and contribute to the understanding and development of an effective vaccine remains necessary.

Prophylactic vaccines in humans would be a very effective control method for Chagas disease, which would also be further improved if they were also used in the veterinary sectors.

The therapeutic use of DNA vaccines against Chagas disease could improve the effectiveness of current treatment and thus provide a better prognosis for the patient. These vaccines for therapeutic use have shown no adverse or toxic effects.

The prophylactic or therapeutic DNA vaccines have been shown to reduce the duration and signs of the acute stage, whereas in the chronic phase have avoided or lessened the severity of heart damage and thereby prolonged the patients’ lifetime.

Prophylactic vaccines have failed so far to prevent parasitic infection; therefore, the current challenge is to identify the ideal antigen.

There is a need to create new formulations that can improve the existing protection achieved so far, as well as to test new methods of administration.

## Abbreviations

CTL: Cytotoxic T lymphocytes
IL: Interleukin
GM-CSF: Granulocyte-macrophage colony-stimulating factor
Cz: Cruzipain
SCz: *Salmonella*carrying a plasmid coding for cruzipain
rCz: recombinant cruzipain
Ig: Immunoglobulin
IFN-γ: Interferon-gamma
ASP: Amastigote surface protein
NK: Natural killer
α-GalCer: α-galactosylceramide
FCaBP: Flagellar Ca(2+) binding protein
Tcβ3: Homologue of the adaptin AP-3 complex β3 subunit
NFκB: Nuclear factor kappa-light-chain-enhancer of activated B cells
MHC: Major histocompatibility complex
PFR: Paraflagelar rod protein
HSP70: 70 kilodalton heat shock protein
CCL4/MIP-1 beta: Chemokine (C-C mofif) ligand 4/macrophage inflammatory protein 1 beta
TNF: Tumor necrosis factor
CINVESTAV-IPN: Centro de Investigación y de Estudios Avanzados of the Instituto Politécnico Nacional
BIRMEX: Laboratorios de Biológicos y Reactivos de México, S. A. de C. V.
HuAd5: Human adenovirus type 5
